# The State of Art on Co-Morbid Insomnia and Sleep Apnea (COMISA)

**DOI:** 10.3390/brainsci11081079

**Published:** 2021-08-18

**Authors:** Luigi De Gennaro

**Affiliations:** 1 Department of Psychology, Sapienza University of Rome, Via dei Marsi 78, 00185 Rome, Italy; luigi.degennaro@uniroma1.it; 2 IRCCS Fondazione Santa Lucia, Via Ardeatina 306/354, 00179 Rome, Italy

Insomnia and obstructive sleep apnea (OSA) are two prevalent sleep disorders which share nocturnal sleep disturbances, impairments to daytime activity and quality of life, and high healthcare and social costs. The treatment of both disorders is often limited by the relatively low acceptance or long-term adherence to cognitive and behavioral therapy for insomnia (CBTi) or continuous positive airway pressure (CPAP) therapy, respectively.

Unsurprisingly, the review published by Sweetman et al. [1] on comorbid insomnia and apnea (COMISA) is one of the most cited articles in Brain Sciences 2019. This review discussed how important and high the rate of comorbid insomnia and apnea is. According to the authors [1], insomnia was long (actually, too long) considered secondary to apnea. Only recently has COMISA been studied as an independent topic, aiming to clarify how it requires different treatments and also that its response rate is quite low in treatment studies [1]. In fact, after the pioneering Science publication by Christian Guilleminault in 1976 [2], COMISA has largely been neglected for around thirty years. Subsequently, a series of studies raised the attention around COMISA, portraying its prevalence: 35% of insomnia patients have an Apnea–Hypopnea Index of ≥15, which corresponds to moderate sleep apnea, while 38% of OSA patients meet the criteria of insomnia [3]. Furthermore, there is some evidence to suggest that OSA contributes to the exacerbation of insomnia [4]

Based upon recent randomized controlled trials, some issues that should be considered in the future research agenda include [1]:

(1) Refine the diagnosis and measurement of COMISA to assess both the independent and the shared symptoms of insomnia and OSA disorder.
(2) Investigate the potential bi-directional relationships in COMISA in order to identify the predictors of the greatest response to insomnia or OSA treatments.
(3) Examine the efficacy of the combined treatments for COMISA, administering both cognitive and behavioral therapy for insomnia (CBTi) and continuous positive airway pressure (CPAP) therapy.
(4) Explore the mechanism(s) underlying the relationship between obstructive sleep apnea and insomnia.
(5) Define the role of circadian rhythms in COMISA.
(6) Clarify the moderating role of sex, race, age, and regional differences.
(7) Assess the clinical evolution of insomnia during OSA treatments.

Co-morbid insomnia and sleep apnea (COMISA) represent a clear model of bi-directional relationships between two different disorders. The review by Sweetman et al. [1] is able to stimulate further comprehension of these bi-directional relationships and underlying mechanisms, and thus aid in the development of novel and effective treatments.

## References

[1] Sweetman A., Lack L., Bastien C. (2019). Co-morbid insomnia and sleep apnea (COMISA): Prevalence, consequences, methodological considerations, and recent randomized controlled trials. Brain Sci..

[2] Guilleminault C., Eldridge F.L., Dement W.C. (1973). Insomnia with sleep apnea: A new syndrome. Science.

[3] Zhang Y., Ren R., Lei F., Zhou J., Zhang J., Wing Y.-K., Sanford L.D., Tang X. (2019). Worldwide and regional prevalence rates of co-occurrence of insomnia and insomnia symptoms with obstructive sleep apnea: A systematic review and meta-analysis. Sleep Med. Rev..

[4] Sweetman A., Lack L., McEvoy R.D., Smith S., Eckert D.J., Osman A., Carberry J.C., Wallace D., Nguyen P.D., Catcheside P. (2021). Bi-directional relationships between co-morbid insomnia and sleep apnea (COMISA). Sleep Med. Rev..

